# Optical breast cancer margin assessment: an observational study of the effects of tissue heterogeneity on optical contrast

**DOI:** 10.1186/bcr2770

**Published:** 2010-11-05

**Authors:** Stephanie Kennedy, Joseph Geradts, Torre Bydlon, J Quincy Brown, Jennifer Gallagher, Marlee Junker, William Barry, Nimmi Ramanujam, Lee Wilke

**Affiliations:** 1 Department of Biomedical Engineering, Duke University, 136 Hudson Hall, Durham, NC 27708, USA; 2 Department of Pathology, Duke University Medical Center, 200 Trent Drive, Durham, NC 27710, USA; 3 Department of Surgery, Duke University Medical Center, 3116 N Duke Street, Durham, NC 27704, USA; 4 Department of Bioinformatics and Biostatistics, Duke University, 2424 Erwin Rd, Durham, NC 27710, USA; 5 Department of Surgery, University of Wisconsin School of Medicine and Public Health; 600 Highland Avenue CS H4/278, Madison, WI 53792, USA

## Abstract

**Introduction:**

Residual cancer following breast conserving surgery increases the risk of local recurrence and mortality. Margin assessment presents an unmet clinical need. Breast tissue is markedly heterogeneous, which makes distinguishing small foci of cancer within the spectrum of normal tissue potentially challenging. This is further complicated by the heterogeneity as a function of menopausal status. Optical spectroscopy can provide surgeons with intra-operative diagnostic tools. Here, we evaluate *ex-vivo *breast tissue and determine which sources of optical contrast have the potential to detect malignancy at the margins in women of differing breast composition.

**Methods:**

Diffuse reflectance spectra were measured from 595 normal and 38 malignant sites from the margins of 104 partial mastectomy patients. All statistical tests were performed using Wilcoxon Rank-Sum tests. Normal and malignant sites were compared before stratifying the data by tissue type and depth and computing statistical differences. The frequencies of the normal tissue types were separated by menopausal status and compared to the corresponding optical properties.

**Results:**

The mean reduced scattering coefficient, < μ_s_' >, and concentration of total hemoglobin, [THb]), showed statistical differences between malignant (< μ_s_' > : 8.96 cm^-1 ^± 2.24_MAD_, [THb]: 42.70 μM ± 29.31_MAD_) compared to normal sites (< μ_s_' > : 7.29 cm^-1 ^± 2.15_MAD_, [THb]: 32.09 μM ± 16.73_MAD_) (*P *< 0.05). The sites stratified according to normal tissue type (fibro-glandular (FG), fibro-adipose (FA), and adipose (A)) or disease type (invasive ductal carcinoma (IDC) and ductal carcinoma *in situ *(DCIS)) showed that FG exhibited increased < μ_s_' > and A showed increased [β-carotene] within normal tissues. Scattering differentiated between most malignant sites, DCIS (9.46 cm^-1 ^± 1.06_MAD_) and IDC (8.00 cm^-1 ^± 1.81_MAD_), versus A (6.50 cm^-1 ^± 1.95_MAD_). [β-carotene] showed marginal differences between DCIS (19.00 μM ± 6.93_MAD_, and FG (15.30 μM ± 5.64_MAD_). [THb] exhibited statistical differences between positive sites (92.57 μM ± 18.46_MAD_) and FG (34.12 μM ± 22.77_MAD_), FA (28.63 μM ± 14.19_MAD_), and A (30.36 μM ± 14.86_MAD_). The diagnostic ability of the optical parameters was affected by distance of tumor from the margin as well as menopausal status. Due to decreased fibrous content and increased adipose content, normal sites in post-menopausal patients exhibited lower < μ_s_' >, but higher [β-carotene] than pre-menopausal patients.

**Conclusions:**

The data indicate that the ability of an optical parameter to differentiate benign from malignant breast tissues may be dictated by patient demographics.  Scattering differentiated between malignant and adipose sites and would be most effective in post-menopausal women.  [β-carotene] or [THb] may be more applicable in pre-menopausal women to differentiate malignant from fibrous sites.  Patient demographics are therefore an important component to incorporate into optical characterization of breast specimens.

## Introduction

According to the 2009 American Cancer Society statistics, there were an estimated 192,370 individuals diagnosed with invasive breast cancer and 62,280 with *in situ *breast cancer in the United States [1]. Treatment for breast cancer involves a multi-disciplinary approach incorporating surgery (lumpectomy, mastectomy and/or removal of axillary lymph nodes), chemotherapy, directed biologic therapy and/or radiation therapy. Breast conserving surgery (BCS), also known as a lumpectomy or partial mastectomy, is generally considered to be the recommended surgical choice for women with early stage breast cancer (Stages 0, I, II) and for those with Stage II to III disease who undergo successful neo-adjuvant treatment to reduce their tumor burden [2,3]. Breast Conserving Therapy (BCT) refers to BCS followed by radiation therapy. The rate of BCT versus mastectomy is regionally variable and partly dependent on the patients' breast to tumor size ratio. On average, between 50 and 75% of patients with breast cancer receive BCT [4,5]. Traditionally, analyses of multiple randomized clinical trials comparing patients who received BCT to those with mastectomy have shown equivalent long-term survival [6]. Recently, however, the meta-analyses by the Early Breast Cancer Trialists group has shown that for every four women who develop a local recurrence after BCT there is one potential mortality; evidence supporting the need to reduce the risk of local recurrence through complete tumor excision [7,8].

In BCS, the surgeon attempts to excise the cancerous area along with a margin of normal tissue, while conserving as much breast volume as possible. The specimen is viewed as a cube with six margins: anterior, posterior or deep, medial, lateral, inferior and superior. The surgeon, immediately upon removal of the tissue, uses surgical clips and/or sutures to orient the specimen for pathology. The excised tissue and any additional margin shavings are sent to surgical pathology for inking which further delineates the six margins, followed by a thorough microscopic evaluation. At Duke University Medical Center (DUMC), a margin is denoted as positive if malignant cells are found on the ink used by the pathologists; close if malignant cells are found within 2 mm of the margin, and negative if there are no malignant cells within 2 mm of the margin. The final surgical margin status is a determinant of local recurrence, thus patients are advised to have a re-excision if disease is found within 2 mm of the margin [9,10]. The quoted rates of second surgeries vary in the literature and range from 12% to as high as 60% [2,8,10,11].

Pathologic touch preparation cytology and frozen section analyses are used in a select number of hospitals for intra-operative breast margin assessment and have been shown to have limitations, motivating the clinical need for improved intra-operative margin assessment tools [12]. Touch-prep cytology has a sensitivity ranging from 75 to 100% and allows the entire margin to be surveyed but requires an on-site trained pathologist and can only detect tumor cells extending all the way up to the surgical margin; it is unable to identify close margins [9,13,14]. The sensitivity of frozen section analysis ranges from 59 to 91% [15-18]. This technique also requires an on-site trained pathologist, performs poorly with fatty breast tissue and typically samples only a very small portion of the surgical margin [19].

A number of academic [13,20-24] and commercial [25-27] groups have worked on or are developing tools for intra-operative assessment of breast margins. Dune Medical has developed a pen-like probe called the MarginProbe™, which uses radio waves to measure the electromagnetic properties of breast tissue over a 7 mm diameter area with a 1 mm sensing depth. Their device was reported to have a sensitivity and specificity of 70% and 70%, respectively, and varied with the size of cancer features [28].

Pioneering optical studies to characterize breast tumors were carried out by Bigio *et al. *[22] in which they used reflectance spectroscopy in the ultraviolet (UV) to visible (VIS) range to evaluate sites within the tumor bed in 24 patients (13 cancer and 59 normal sites). This work suggested an important role for optical spectroscopy in margin analysis. Keller *et al*. published on the ability of diffuse reflectance and fluorescence spectroscopy to detect cancerous sites on excised breast tumors in 32 patients (145 normal and 34 individual tumor sites), and reported a sensitivity and specificity of 85% and 96%, respectively, for classifying individual sites (not margins) [24]. Volynskaya *et al*. used modified diffusion theory and multivariate curve resolution, to extract absorption and scattering information from 104 reflectance and fluorescence spectra in breast biopsies from 17 patients to achieve a sensitivity and specificity of 100% and 96% [29]. Haka *et al*. utilized Raman spectroscopy from tumor sites on freshly sliced lumpectomy specimens in 21 patients (123 normal and 6 malignant tissue sites) and exploited fat and collagen contrast to achieve a sensitivity and specificity of 83% and 93%, respectively for classifying individual sites [23]. Nguyen *et al. *[20] demonstrated that optical coherence tomography detects *ex-vivo *margin positivity in 20 patients (11 positive/close margins and 9 negative margins), with a sensitivity and specificity of 100% and 82%, respectively by exploiting scattering associated with increased cell density.

Our group recently published the results of a quantitative diffuse reflectance spectroscopy technique to non-destructively image entire lumpectomy margins in 48 patients [8,30]. Successive placement of a multi-channel probe samples an area of approximately 2 × 4 cm, with 5 mm resolution (approximately the thickness of slices cut for pathology) and sensing depth of 0.5 to 2.2 mm (450 to 600 nm) (on a par with the pathologic criterion for clear margins) [31]. The diffuse reflectance spectra per site were analyzed with a feature extraction algorithm based on a fast, scalable Monte Carlo model developed by our group [32,33] to quantitatively determine absorption and scattering contrast in the breast. The sources of contrast were used to create tissue composition maps and then were applied to a decision-tree model to differentiate positive from negative margins. This margin level approach resulted in a sensitivity and specificity of 79.4% and 66.7% respectively on 55 margins from 48 patients [8,31].

Normal breast tissue is markedly heterogeneous, which makes distinguishing small foci of cancer within the spectrum of normal tissue potentially challenging. This is further complicated by the heterogeneity as a function of menopausal status. The goal of the work presented here was to evaluate the site level optical properties of pathologically confirmed normal and malignant *ex-vivo *human breast tissue sites, and to determine which sources of optical contrast have the potential to detect malignancy on margins in women of differing normal and malignant breast composition. Our data show how *ex-vivo *optical properties of positive and close sites on breast tumor margins are strongly dependent on breast tissue composition and patient demographics, which should be accounted for in developing classification algorithms. The results of this paper will provide an understanding of the optical contrast from individual pixels which will be informative for both a site level and margin level method of margin assessment.

## Materials and methods

### Clinical protocol

An *ex-vivo *study using diffuse optical spectroscopy to evaluate partial mastectomy specimens in patients undergoing surgery for breast malignancies was approved by the Institutional Review Board at Duke University as detailed in previous publications [8,31]. Informed consent was obtained from eligible patients undergoing a partial mastectomy (lumpectomy). The surgeries were performed on 104 patients by one of five collaborating surgeons at the Duke University Ambulatory Surgery Center. The optical study was performed post excision and did not alter the standard operating procedures. The surgeons performed sentinel lymph node dissection in addition to the partial mastectomy on a subset of patients. For sentinel lymph node dissection, Lymphazurin™ (United States Surgical, a division of Tyco Healthcare Group LP, Norwalk, CT, USA) was injected into the subareolar or peri-tumoral area in order to identify the draining lymph nodes for the respective cancers. In these cases, the Lymphazurin™ was also found within the lumpectomy margins in varying degrees, the presence of which was recorded for each consented patient. Patient demographics were recorded, including menopausal status. Women were designated as post-menopausal if they had one of the following: either a bilateral salpingo-oopherectomy or lack of a menstrual cycle for greater than one year.

**Figure 1:**
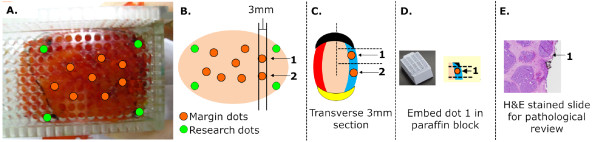
**Photograph of an actual margin after imaging and pathological methodology**. **(A) **The six orange dots provide the location of inked sites and the four green dots denote the boundaries of the margin used for pathologic co-registration and diagnoses. Four margin dots (green) denote the corners of the measured margin while the eight orange dots denote the research dots **(B)**. A transverse slice is taken from the specimen with the margins inked for orientation **(C)**. Each research dot is embedded in a paraffin block from routine processing **(D)**. A 5 μm section is taken from the paraffin block, stained and examined under a microscope by a specialized breast pathologist **(E)**.

In each partial mastectomy case, the surgeon removed the specimen, then placed clips and sutures to orient the specimen for pathological assessment. This clinical protocol and margin level pathologic processing is described in more detail in a previous publication by Wilke *et al. *[8]. Diffuse reflectance (DR) spectral imaging was performed on the margins of the lumpectomy specimens between 10 and 20 minutes after excision. Margins were selected based on specimen mammograms and surgeon recommendations for potentially positive margins. Typically, spectroscopic data were measured from one margin per case and when time permitted, measured from up to five margins before the end of the operation. For a more detailed analysis of tissue contrast with respect to different breast tissue types, an additional 6 to 10 sites were randomly inked orange (Figure 1A) on one of the imaged margins. In a few instances, sites were inked on two margins. Tissue composition at those specific sites was determined via a separate microscopic evaluation by the study pathologist (JG). The margins with inked research dots were processed as seen in Figure 1 and as described in the *Histopathology *section below.

Of the 854 sites from 105 margins (104 patients), the pathologist was unable to microscopically identify the inked site to generate a diagnosis for 91 sites. From the remaining 763 sites, 94% (715) were normal and 6% (48) were positive or close for malignant disease. Seventeen percent (120) of the normal sites and 2% (10) of the malignant sites were excluded. Reasons for exclusion included excess Lymphazurin™, a missing diagnosis, unknown distance of the tumor from the margin, or poor optical signal to noise resulting in extreme outliers which were more than three times the inter-quartile range (IQR) below the first quartile or above the third quartile of all sites. Sites were considered to have too much Lymphazurin™ if intensity of the diffuse reflectance spectrum at 600 nm was lower than the intensity at 450 nm, indicating that Lymphazurin™ overwhelmed the hemoglobin absorption. Removing these samples resulted in 595 normal sites and 38 malignant sites from 101 margins (100 patients) retained for the final analysis. The first stage of the analysis to show high level tissue contrast was performed between all malignant and normal sites from 101 margins (100 patients). The second stage of the study to look at the effect of tissue type and disease depth examined only the retained sites with a predominant tissue type as described below in *Histopathology*; this excluded one patient and one margin that contained heterogeneous sites leaving 100 margins from 99 patients (32 malignant and 408 normal sites). Patients without a known menopausal status or who were peri-menopausal (seven margins, six patients) were excluded from the third stage of the analysis which only included pre- and post-menopausal normal samples. This resulted in 94 margins from 93 patients (553 normal sites).

### Histopathology

Routine histopathology was performed on each of the specimens. In Figure 1A, B, the green margin dots and orange research dots are shown on a margin. To obtain the site level histology, each specimen underwent transverse sectioning into 3 mm thick slices. Figure 1C shows a 3 mm section containing two research dots (1 and 2). The transverse slice has ink on the borders corresponding to the specimen's surgical orientation. Each slice was further sectioned so that a dot was contained in an approximately 20 × 10 × 3 mm piece of tissue, which was then embedded in paraffin wax (Figure 1D). A 5 μm thick slice was then taken from the embedded specimen, stained with hemotoxylin & eosin (H&E) and microscopically evaluated by the study pathologist (JG) (Figure 1E). A site level diagnosis was generated for each inked location. Because the theoretical sensing depth of the probe ranged from 0.5 to 2.2 mm (see *Clinical measurements of diffuse reflectance spectra*), the study pathologist also provided a quantitative measure of the distance of malignancy from the margin ranging from 0 mm to 2 mm. Malignant sites were classified by the distance of malignant cells from the surgical margin ("positive" = cancer at surface, "close" = between 0 to 2 mm from the surface) as well as the type of cancer present. For 'close' sites, the actual distance of the malignant cells from the surgical margin was recorded. The type of cancer was specified as IDC, invasive lobular carcinoma (ILC), DCIS, or lobular carcinoma *in situ *(LCIS). Normal tissues were classified according to the predominant tissue type present. Due to the heterogeneity of breast tissue, the normal sites were broken down into fibro-glandular (FG), fibro-adipose (FA), adipose (A), fibro-cystic change (FCC), fibrous (F), fat necrosis (FN), or sites with mixed tissue types (Mx). Samples with a predominant tissue type and unusually prominent vessels or hemorrhage were categorized as vessels (V).

### Clinical measurements of diffuse reflectance spectra

This study utilized instrumentation from Jobin-Yvon HORIBA (Edison, NJ, USA) that included a 450 W Xenon arc lamp, a double excitation monochromator (Gemini 180, JY HORIBA), an imaging spectrograph (TRIAX 320, Jobin-Yvon HORIBA) and a Peltier-cooled CCD (Symphony, Jobin-Yvon HORIBA). This study was performed using an in-house designed multi-channel fiber-optic probe manufactured by RoMack, Inc. (Williamsburg, VA, USA). The multi-channel probe had eight individual channels, where each channel consisted of a core of 19 hexagonally packed illumination fibers and 4 collection fibers arranged at the corners of the illumination core. All fibers were 200 μm in diameter with a numerical aperture of 0.22. The breast specimen, once excised, was oriented in a Plexiglass™ box using the clips and sutures to maintain orientation with the surgeon and pathology. The channels of the probe were then interfaced with the Plexiglass™ box via an aluminum adaptor. The holes in the Plexiglass™ box were spaced 5 mm apart (center-to-center), which means that the tissue in the box was imaged with a 5 mm spatial resolution. Diffuse reflectance spectra were measured simultaneously from eight holes (or pixels) for each placement of the multi-channel probe in the Plexiglass™ box. To ensure that the entire margin was sampled, the multi-channel probe was manually moved over the surface of the specimen such that one spectrum was eventually obtained from each hole of the box. To ensure that there was no optical cross-talk between adjacent probes during any single measurement of eight pixels, the channels were arranged in a 4 × 2 array with a center-to-center spacing of 10 mm for each channel. This overall procedure ensured that data were collected uniformly and repeatedly over the tissue surface, with minimal cross-talk between channels. The average margin was covered with 64 holes (or pixels), which means that on average the entire margin surface was sampled with eight placements of the multi-channel probe. A custom LabVIEW (National Instruments Corporation, Austin, TX, USA) application was used to control data collection and keep track of the original spatial locations of each diffuse reflectance measurement on the tissue surface, so that accurate spatial maps of the tissue could be reconstructed after completion of imaging. The imaging procedure is described in detail in our previous publications [8,31,34]. After imaging, 6 to 10 tissue sites (or pixels) were randomly chosen and inked with a wooden dowel inserted through the Plexiglass™ box holes for the detailed pathological analysis described in *Histopathology*. The sensing depth of the multi-channel probe was previously simulated. These simulations were carried out using a weighted photon Monte Carlo model, previously described by Liu *et al. *[35] and Zhu *et al. *[36]. The theoretical sensing depth was determined using the weighted visiting frequency as a function of depth as described by Bydlon *et al. *[31]. Based on the simulations over the wavelength range of 450 to 600 nm, the sensing depth of the multi-channel probe varied with tissue type: 0.5 to 1.5 mm in positive tissues, 0.7 to 2.2 mm in adipose tissues, and 0.6 to 1.5 mm in fibro-glandular tissue [31].

We have also evaluated the reproducibility and accuracy of these diffuse reflectance measurements. Bydlon *et al*. reported the coefficient of variation (σ/μ) calculated from 10 repeated measurements for the system [31]. The median coefficient of variation was calculated and was found to be less than 0.11 for all extracted parameters (β-carotene, < μ_s_' >, THb, β-carotene/< μ_s_' >, THb/< μ_s_' >, and β-carotene/THb) indicating little deviation from the mean in all measurements. Using tissue mimicking phantoms, accuracy for determining the underlying tissue compositional features was found to be 5.57 ± 3.89% for [THb], 14.99 ± 13.6% for Crocin (similar absorption to [β-carotene]), and 9.81 ± 6.89% for < μ_s_'> [31]. These errors in extraction accuracy for [THb], [Crocin] and < μ_s_'> were less than the percent difference observed between positive vs. adipose tissue; the errors in [THb] and < μ_s_'> were less than the percent difference observed between positive vs. fibro-glandular tissue [31].

After spectral imaging was completed, calibration measurements were made on a Spectralon reflectance standard (Labsphere, North Sutton, NH, USA) to account for the wavelength dependence and throughput of the system, as described in greater detail in previous studies [8,36,37]. The spectral imaging system is also further described in previous publications by our group [31,34,38].

### Extraction of tissue optical properties

Optical properties of the measured tissue were extracted from the diffuse reflectance spectra using a fast, scalable Monte Carlo inverse model [33]. The Monte Carlo model was used to analyze the diffuse reflectance spectra from 450 to 600 nm. The strongest intrinsic absorbers in this range were assumed to be oxygenated and de-oxygenated hemoglobin, as well as β-carotene. Lymphazurin™ was included as an extrinsic absorber in the model as previously described [39]. An arbitrary absorber was included by modeling the extinction as a Gaussian with a center mean wavelength of 515 nm and a standard deviation of 10 nm, and was used to account for the difference in shape between the tabulated absorption of β-carotene and the absorption of β-carotene in tissue [32,39]. This deviation in fit was seen between 500 to 530 nm. The magnitude of the Gaussian function was previously found to be highly correlated (Pearson's correlation coefficient of 0.9) to [β-carotene]. In this previous study, the addition of the Gaussian function improved the fits but did not significantly affect the conclusions regarding the extracted parameters when compared to the case where the Gaussian absorber was not used [32]. The concentrations of β-carotene, oxygenated hemoglobin, de-oxygenated hemoglobin, and Lymphazurin™were extracted using the model. Other parameters such as hemoglobin saturation and THb were derived from the concentrations of oxygenated and de-oxygenated hemoglobin. The wavelength dependent reduced scattering coefficient, μ_s_' (λ), was extracted from the model and the mean wavelength-averaged value, < μ_s_' >, was calculated to describe the scattering properties of the probed tissue volume.

### Statistical analysis

The distributions of the optical properties were empirically summarized by the median, median absolute deviance (MAD), IQR, and Tukey whiskers as 1.5*IQR. Optical properties were first compared between all malignant and normal sites from the 101 retained margins. The optical properties were then stratified according to the predominant tissue type and pair wise comparisons were made using Wilcoxon Rank-Sum tests and applying a Bonferroni correction factor for multiple comparisons. The Inverse Monte Carlo model, statistical comparisons and figures were generated using MATLAB™ (MatWorks Inc., Natick, Massachusetts, USA).

## Results

The primary aim of this study was to evaluate the VIS optical properties of normal and malignant breast tissues to understand the effect of the normal tissue types, different disease types, depth of disease, and patient demographics on the extracted optical properties. The 38 malignant samples were comprised of IDC (*n *= 22), DCIS (*n *= 12), LCIS (*n *= 3), and ILC (*n *= 1) (Table 1). The malignant sites at 0 mm are considered positive by standard pathology; the sites from 0^+ ^to 1 mm or 1^+ ^to 2 mm are considered pathologically "close". The malignant sites were separated by distance from the margin as follows: on ink at 0 mm (*n *= 10), 0^+ ^to 1 mm of the margin (*n *= 17), 1^+ ^to 2 mm from the margin (*n *= 11). The 595 normal sites were divided into adipose (A) (*n *= 324), mixed tissue samples (Mx) (*n *= 112), vessel samples (V) (*n *= 64), fibro-adipose (FA) (*n *= 60), fibro-glandular (FG) (*n *= 24), fibrocystic change (FCC) (*n *= 4), fibrous (F) (*n *= 6), and fat necrosis (FN) (*n *= 1) as shown in (Table 1). Of these normal samples, 142 were from pre-menopausal patients and 411 were from post-menopausal patients.

**Table 1:** Classification of the *ex-vivo *sites for normal and malignant tissue types

Normal		595	Malignant		38
	Adipose (A)	324	0 mm		10
	Mixed tissue types (Mx)	112		IDC	8
	Fibro-adipose (FA)	60		DCIS	2
	Vessels (V)	64	0^+ ^to 1 mm		17
	Fibro-glandular (FG)	24		IDC	9
	Fibrous (F)	6		DCIS	5
	Fibro-cystic change (FCC)	4		ILC	1
	Fat Necrosis (FN)	1		LCIS	2
			1^+ ^to 2 mm		11
				IDC	5
				DCIS	5
				LCIS	1

The malignant sites are stratified according to disease type (IDC, DCIS, ILC, or LCIS) and by depth. Malignant sites at 0 mm are considered positive; sites from 0^+ ^to 1 mm or 1^+ ^to 2 mm are considered close. DCIS, ductal carcinoma *in situ*; IDC, invasive ductal carcinoma; ILC, invasive lobular carcinoma; LCIS, lobular carcinoma *in situ*.

### Optical contrast: malignant vs. normal

**Figure 2:**
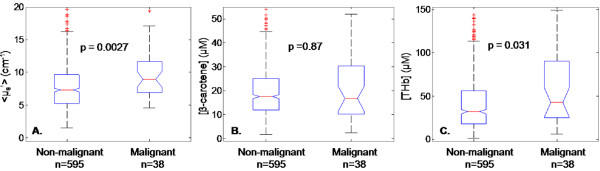
**Boxplots of the extracted parameters for malignant versus non-malignant tissues**. **(A) **< μ_s_' >, **(B) **β-carotene concentration, and **(C) **total hemoglobin concentration. To improve visualization of the distributions of the extracted parameters, the boxplots were scaled to a maximum of 20 cm^-1 ^for < μ_s_' >, 50 μM for [β-carotene] and 150 μM for [THb]. The *P*-values are displayed for the Wilcoxon Rank Sum tests.

Optical properties were compared between normal and malignant sites and the results are shown in Figure 2. The malignant sites were comprised of all "positive" and "close" (0 to 2 mm) sites representing several disease types (IDC, DCIS, ILC, and LCIS), and the normal sites included the entire 595 normal tissue types listed in Table 1. Scattering, < μ_s_' >, was significantly increased in malignant sites (8.96 cm^-1 ^± 2.24_MAD_) compared to normal sites (7.29 cm^-1 ^± 2.15_MAD_) (*P *= 0.0027, Figure 2A). Furthermore, [THb] was also significantly increased in malignant sites (42.70 μM ± 29.31_MAD_) compared to normal (32.09 μM ± 16.73_MAD_) sites (*P *= 0.031, Figure 2C). However, [β-carotene] could not differentiate malignant tissues from benign tissues (*P *= 0.87, Figure 2B).

### Optical contrast: normal heterogeneity

**Figure 3:**
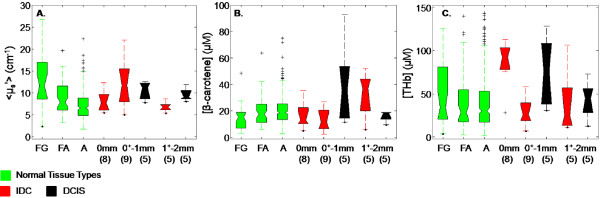
**Boxplots of (A) < μ_s_' >, (B) [β-carotene], and (C) [THb] for normal and malignant tissue subcategories**. Normal tissue consists of fibro-glandular (FG), fibro-adipose (FA), adipose (A); malignant tissue consists of invasive ductal carcinoma (IDC) and ductal carcinoma *in situ *(DCIS). All malignant tissues were stratified according to depth: positive (0 mm), close within 1 mm (0^+ ^to 1 mm), close between 1 and 2 mm (1^+ ^to 2 mm). DCIS (0 mm) is not shown due to its small sample size (*n *= 2).

Mixed tissues and vessels were excluded from this analysis because the fractional composition of adipose, glands, and fibrous tissue within each site was not specified by pathology. Fibrous, fibrocystic change, and fat necrosis sites were excluded due to their respective small sample sizes. The Wilcoxon-rank sum *P*-values were corrected for three comparisons. In Figure 3, the normal sites stratified according to the predominant tissue type showed that < μ_s_'> increased with increased fibrous content. All three predominant tissue types showed statistically significant scattering differences from one another (FG v. A, adj. *P *< 0.0001; FG v. FA, adj. *P *= 0.011; A v. FA, adj. *P *= 0.0026). The median scattering and median absolute deviance for the normal tissue types were as follows: FG (11.61 cm^-1 ^± 3.44_MAD_), FA (7.80 cm^-1 ^± 2.86_MAD_), and A (6.50 cm^-1 ^± 1.95_MAD_). [β-carotene] was higher in adipose (adj. *P *= 0.017) than in fibro-glandular sites. Marginal differences in [β-carotene] are noted between FG and FA (nominal *P *= 0.045), but do not reach statistical significance after accounting for multiple comparisons. The resulting median [β-carotene] values were as follows: FG (15.30 μM ± 5.64_MAD_), FA (17.45 μM ± 6.88_MAD_), and A (18.75 μM ± 5.74_MAD_). The concentration of total hemoglobin, [THb], did not show statistical differences between FG (34.12 μM ± 22.77_MAD_), FA (28.63 μM ± 14.19_MAD_), or A (30.36 μM ± 14.86_MAD_).

### Optical contrast: malignant vs. normal variants

This portion of the study focused on a two factor analysis of disease type and disease depth, the data for which is shown in Figure 3. Because normal sites showed a range of values for each parameter (< u_s_' >, [THb], [β-carotene]), we also wanted to examine the range of values obtained from different malignant tissue types, such as IDC and DCIS. The resulting *P*-values were adjusted by accounting for six comparisons. Figure 3 shows the normal variants fibro-glandular (FG), fibro-adipose (FA), and adipose (A) in green, as well as the malignant variants (IDC in red and DCIS in black separated by disease depth). ILC and LCIS were excluded due to the small sample size. For the first part of the analysis, all IDC depths were combined and also all DCIS depths were combined (combined depths are not shown in Figure 3). Overall, FG exhibited the highest median scattering, followed by DCIS (9.46 cm^-1 ^± 1.06_MAD_), IDC (8.00 cm^-1 ^± 1.81_MAD_), FA, then A. The highest median [β-carotene] was observed in DCIS (19.00 μM ± 6.93_MAD_), followed by A, FA, FG then IDC sites (13.89 μM ± 8.29_MAD_). Comparing the concentrations of total hemoglobin across these different normal and disease tissue types resulted in DCIS exhibiting the highest median value (57.42 μM ± 21.58_MAD_), followed by IDC (38.89 μM ± 26.15_MAD_), FG, A and FA. Scattering, < μ_s_' >, could statistically differentiate between IDC and A (adj. *P *= 0.035), and DCIS and A (adj. *P *= 0.0012). [THb] was able to separate DCIS from A (adj. *P *= 0.029). While several parameters could not distinguish between specific variants when applying the Bonferroni correction for multiple comparisons, unadjusted *P*-values did show several additional differences among variants. For example, scattering was observed to be higher in FG than in IDC with an unadjusted *P*-value of *P *= 0.044; DCIS exhibited increased [β-carotene] compared to FG with an un-adjusted *P*-value of *P *= 0.028. [THb] was also increased in DCIS compared to FA with an unadjusted *P*-value of *P *= 0.023.

The second factor in the analysis involved evaluating how optical properties varied with depth in order to determine the contribution of the "intervening" normal tissue to the optical evaluation. The type of normal tissue between the margin and malignancy was not specifically identified by the pathologist in this study. The disease types are shown in Figure 3 as positive sites (0 mm), close sites within 1 mm of the margin (0^+ ^to 1 mm), and close sites between 1 to 2 mm from the margin (1^+ ^to 2 mm). Positive DCIS sites (0 mm) are not shown in Figure 3 due to the small sample size (*n *= 2). For this analysis, IDC and DCIS samples were combined and grouped according to depth of malignancy as the sample size was not large enough to statistically evaluate optical parameters by both disease type and depth. This grouping of IDC and DCIS according to depth of malignancy showed that < μ_s_'> was highest within the close sites (0^+ ^to 1 mm) (11.41 cm^-1 ^± 2.61_MAD_), followed by positive sites (0 mm) (8.60 cm^-1 ^± 1.33_MAD_), and close sites (1^+ ^to 2 mm) (8.31 cm^-1 ^± 1.52_MAD_). In contrast, [β-carotene] followed an increasing trend with increased distance of malignancy; it was highest in close sites (1^+ ^to 2 mm) (19.00 μM ± 7.81_MAD_), followed by close sites (0^+ ^to 1 mm) (15.59 μM ± 9.53_MAD_), then positive sites (0 mm) (13.89 μM ± 6.23_MAD_). Lastly, the concentration of total hemoglobin was found to decrease as follows: positive (92.57 μM ± 18.46_MAD_), close (1^+ ^to 2 mm) (36.98 μM ± 18.24_MAD_), and close (0^+ ^to 1 mm) (36.08 μM ± 12.81_MAD_). [THb] showed statistical significant differences between positive sites (0 mm) and FG (un-adj. *P *= 0.0069), FA (adj. *P *= 0.0037) and A (adj. *P *= 0.0004), as well as between close sites (0^+ ^to 1 mm) and A (*P *= 0.0036) when corrected for nine comparisons. The other optical parameters did not show differences that were statistically significant after accounting for multiple comparisons. However, the trends demonstrate that the median and MAD of the optical parameters were affected by the depth of disease and the unknown intervening tissue. The depth analysis shows that depth plays an important role but due to the small sample size it is not possible to specifically indicate how depth affects each disease type.

### Optical contrast: menopausal status and normal variants

A total of 45 optical sites were excluded from this part of the analysis due to the patients having an undefined menopausal status (lack of menses in patients younger than 40 years of age; peri-menopausal or unknown menopausal status). The malignant sites could not be analyzed by menopausal status because of the low number of malignant sites from pre-menopausal patients.

**Figure 4:**
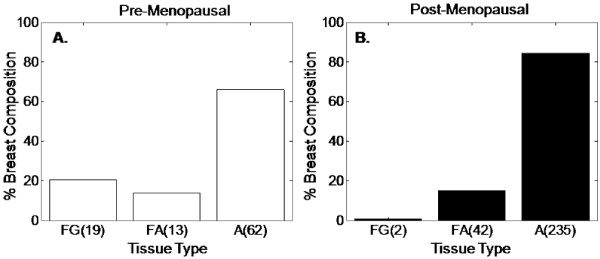
**Histograms of the percent composition of the three main normal tissue types**. The fibro-glandular (FG), fibro-adipose (FA) and adipose (A) sites were stratified by **(A) **pre-menopausal sites and **(B) **post-menopausal sites.

The relative frequency of adipose tissue is higher in post-menopausal patients while the relative frequency of fibro-glandular tissue is higher in pre-menopausal patients. The histograms in Figure 4 represent A) the percentage of the sites from pre-menopausal patients that were composed of FG, FA and A sites, and B) the percentage of the sites from post-menopausal patients that were composed of the same three tissue types. Regardless of menopausal status, adipose tissue was shown to be the most likely tissue type on a margin accounting for 80% of the combined total 373 sites. However, differences were observed in the relative contributions of normal tissue types between pre- and post-menopausal patients. The post-menopausal women showed 84% adipose sites, 15% fibro-adipose sites and 1% fibro-glandular sites. Pre-menopausal women, however, showed 66% adipose sites, 14% fibro-adipose sites, and 20% fibro-glandular sites.

In order to verify that the measured optical variability was due to differences in the relative amounts in tissue composition and not inherent differences in tissue optical properties with menopausal status, we compared the optically measured parameters in purely adipose sites between pre- and post-menopausal patients. The optical parameters from pure adipose sites were compared between pre- and post-menopausal patients and neither scattering (Pre-menopause: 7.54 cm^-1 ^± 2.48_MAD_, Post-menopause: 6.27 cm^-1 ^± 2.36_MAD_) nor [β-carotene] (Pre-menopause: 18.50 μM ± 7.39_MAD_, Post-menopause: 18.77 μM ± 7.613_MAD_) showed statistical differences between pre- and post-menopausal adipose sites. [THb], however, was lower in post-menopausal adipose sites which may be in part to differences in specimen bleeding or decreased overall blood volume (Pre-menopause: 44.16 μM ± 22.06_MAD_, Post-menopause: 27.83 μM ± 21.67_MAD_).

**Figure 5:**
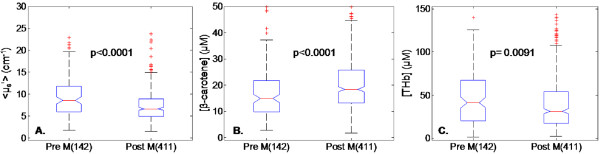
**Boxplots of the extracted parameters from normal sites (*n *= 553)**. The optical properties **(A) **< μ_s_' >, **(B) **[β-carotene], and **(C) **[THb] previously shown in Figure 2 now stratified according to menopausal status. Post-menopausal patients exhibited lower < μ_s_'> and [THb] (panels A. and C.) but higher [β-carotene] (panel C.) than pre-menopausal patients.

The comparison of all normal sites from pre-versus post-menopausal patients showed decreased scattering (*P *< 0.0001) (Figure 5A), increased [β-carotene] (*P *< 0.0001) (Figure 5B), and decreased [THb] (Figure 5C) (*P *= 0.0091) in post-menopausal sites (< μ_s_' > : 6.62 cm^-1 ^± 2.60_MAD_, [THb]: 31.43 μM ± 24.16_MAD_, and [β-carotene]: 18.38 μM ± 8.42_MAD_) compared to pre-menopausal sites (< μ_s_' > :8.5 cm^-1 ^± 3.49_MAD_, [THb]: 41.39 μM ± 26.97_MAD_, and [β-carotene]: 4.84 μM ± 7.72_MAD_). This was consistent with increased fibrous content in pre-menopausal women and increased adipose content in post-menopausal women. Thus, the trends for scattering and [β-carotene] seen in normal sites from pre- and post-menopausal further confirmed the predominant tissue types suspected with each demographic category.

## Discussion

There are two ways to approach the problem with accurate intra-operative breast margin assessment: a whole margin-level method where summary measures are created to describe multiple pixels on a margin and secondly, a site-level approach using pathologically confirmed individual pixels. We have previously published accuracy results on the margin-level classification. This paper is aimed at understanding the optical contrast from individual pixels which will be informative for both methods of assessment. We hypothesized that the heterogeneity of normal breast tissues, the distance of malignant cells from a margin and menopausal status would each influence the interpretation of optical measurements of changes within a breast specimen. Despite the heterogeneity of normal tissue composition, significant differences between all malignant and normal tissues were observed for < μ_s_'> and [THb]. Both of these optical parameters were increased in malignant sites compared to normal sites. Several studies have seen increased scattering in malignant tissues compared to normal tissues [32,36,39,40]. Pogue *et al*. determined that total hemoglobin-based contrast can be up to 200% higher in cancer relative to the normal background [41]. Our data demonstrated consistency with these previous studies for < μ_s_'> and [THb]. [β-carotene] did not differentiate between benign and malignant sites in our study in contrast to Volynskaya *et al*., who found [β-carotene] in bread-loafed specimens (rather than margins) to be higher in normal (fattier) tissues compared to that in tissues with invasive carcinoma [29]. Tissue type has been previously shown to affect optical properties. Breast tissue is heterogeneous such that *ex-vivo *sites will have very different tissue composition depending on the number of malignant cells within an examined volume. The prevalence or probability of seeing a certain tissue type can be related to patient demographics. Thus, we hypothesized that optical contrast could be modulated by tissue composition, distance from the margins and patient demographics, specifically menopausal status.

Scattering variations in normal tissue: our analysis showed that scattering increased as fibrous content increased and adipose content decreased, within normal tissue types consistent with previous studies. Scattering in normal tissues has previously been shown to be negatively correlated with the amount of adipose tissue present [36]. Srinivasan *et al*. found that adipose tissues had low scattering power in the near infrared spectrum (NIR) due to large scatterers, while glandular or fibrous (or by extension fibro-glandular) tissues had high scattering power resulting from more cellular and collagen-based structures [42]. Fibro-glandular tissue is composed of fibroblasts, collagen and elastin bundles, interspersed with ducts/lobules and minimal adipose tissue. In contrast, adipose tissue is comprised of many adipocytes containing lipid droplets with the nucleus flattened along the periphery of the cell resulting in fewer scattering events. Fibro-adipose tissue is a combination of adipocytes and collagen bundles so the resulting scattering falls between the values seen for fibrous and adipose tissue separately. These three normal tissue types exhibited < μ_s_'> values that were significantly different from one another; in summary < μ_s_'> describes the presence or absence of fibrous tissue which, in turn, will be related to a patient's demographic factors.

β-carotene and hemoglobin in normal tissues: [β-carotene] has previously been positively correlated with adipose tissue [36]. [β-carotene] in this study, was also found to increase along with an increasing amount of adipose tissue (that is, from fibro-glandular, to fibro-adipose, to adipose). Hemoglobin volume was found to be decreased in adipose tissue compared to other normal tissue types [43-45]. The trends seen in our study indicated a slight decrease in [THb] for adipose compared to fibro-glandular tissues but the comparisons were not statistically significant. [β-carotene] may provide more information related to the amount of adipose tissue in normal tissues, but [THb] is less susceptible to variations in normal tissue types and will most likely only be informative for distinguishing very vascular malignant sites from avascular normal sites.

The sources of underlying optical contrast were first examined across the normal and malignant variants inclusive of all disease depths. Diagnosing malignant tissues: [THb] and < μ_s_'> exhibited the greatest differences between malignant (IDC and DCIS) and adipose tissues, while [β-carotene] only showed statistical differences between normal tissue types. Prior to correcting the p-values for multiple comparisons, < μ_s_'> was statistically lower in IDC compared to FG with and DCIS exhibited statistically higher [β-carotene] than FG and statistically higher [THb] compared to FA. [THb] was still not statistically higher in IDC than FG since FG is not as avascular as A. The correction factor can cause observed true differences to be rejected; thus we believe that [β-carotene] and [THb] in additional to < μ_s_' >, are worth pursuing further for margin assessment. Distinguishing malignant tissue from normal variants is a clinical challenge due to the known heterogeneity in tumors and the normal micro-environment (pathology). Histologically, IDC presents itself as cancerous cells that have invaded through the basement membrane into the surrounding stroma. These cancerous cells are also surrounded by a fibro-vascular matrix that displaces adipocytes. Because FG (and often FA) tissues can exhibit fibrous and vascular structures, it may be difficult to differentiate IDC from FG or FA based on < μ_s_'> alone as reflected by the statistical findings. [β-carotene] is not a source of contrast either since both IDC and FG have minimal adipose tissue content. [THb] cannot differentiate IDC from FG most likely because both tissue types are well vascularized and because IDC includes all depths of disease. DCIS is comprised of a combination of malignant cells, a fibrous component, and adipocytes. Because of the surrounding normal tissue, DCIS could not be discriminated from FG or FA sites using < μ_s_'> as mentioned above. For the same reasons, it is also difficult to differentiate DCIS from adipose tissue using [β-carotene]. However, it is possible to use [β-carotene] to separate DCIS from FG as it is possible to differentiate DCIS from adipose tissues using < μ_s_' >. The optical contrast for DCIS and IDC compared to the normal variants were similar with the exception of FG; DCIS showed differences from FG using [β-carotene] but none of the parameters separated IDC from FG. This lack of contrast between IDC and FG is a function of not only tissue type but also the depth of disease and the intervening normal tissue.

When distance from the margin was included in the analysis, it became apparent that the distance of intervening normal tissue impacted the extracted optical properties. The trends indicated that scattering increased and [β-carotene] decreased in all close (0^+ ^to 1 mm) compared to positive (0 mm) sites which suggests of the presence of intervening fibrous tissue. In contrast, [β-carotene] was higher while scattering was lower in close sites (1^+ ^to 2 mm) compared to close sites (0^+ ^to 1 mm), indicative of fatty tissue. [THb] was found to be the highest in positive tissues. For sites with close margins, the contrast will be dependent on the intervening normal tissue. The type of intervening normal tissue will be related to the demographic factors. For positive margins however, [THb] shows potential for separating positive sites (IDC and DCIS) from FA and A, as well as from FG. It is clear that the amount and type of normal tissue present between the margin and the malignant cells impacts optical contrast. The complex heterogeneity of intervening tissue increases the difficulty of separating malignant from normal tissues.

Our results show that the frequency and predominance of tissue types on a margin is related to a patient's menopausal status. Post-menopausal patients exhibited a higher frequency of adipose (A) sites and a lower frequency of fibro-glandular (FG) sites compared to pre-menopausal patients. Pre-menopausal women are expected to have denser breast tissue, while post-menopausal women typically have fattier breasts. Cerussi *et al*. looked at the optical properties of breast tissue in the NIR and found pre-menopausal women had higher scattering values and concentrations of THb, but lower lipid content ([β-carotene]) compared to post-menopausal subjects [46]. The trends in this study are consistent with the results observed previously. Suzuki *et al*. studied the optical properties of 30 Japanese women and reported a strong negative correlation of absorption and scattering properties with age, BMI and menstrual status [43]. Cubeddu *et al*. reported changes with age, which could be attributed to tissue fat content [47]. All normal sites were found to demonstrate increased [β-carotene] and decreased < μ_s_'> in post-menopausal patients relative to scattering in pre-menopausal patients.

As shown in this study, menopausal status does not affect the optical properties of individual tissue types, but rather the distribution of tissue types seen as a whole. The site level analysis demonstrated that < μ_s_'> would be the best variable for differentiating cancer against a fatty background and [β-carotene] would be useful in separating DCIS from fibrous tissues. Due to tissue vascularity, malignant tissues with significant vasculature or angiogenesis will show the best contrast with [THb]. For positive margins, [THb] would be effective in separating positive sites (IDC and DCIS) from FG, FA and A. Thus, [THb] has potential for discriminating malignancy at the margin from a fibrous background. For margin assessment, it is necessary to discriminate cancer from the surrounding background; we have demonstrated that a patient's menopausal status will help define the surrounding normal variants allowing the correct optical variable to be applied.

In summary, it is important to understand the underlying sources of contrast when using optical spectroscopy for applications to breast tissue. Our results indicate that steps need to be taken to utilize a patient's demographics to individualize care and optical evaluation. The normal variants on a margin have the potential to decrease the sensitivity and specificity of optical techniques for identifying malignancy. Tailoring optical evaluation to the demographics of a patient may provide a means to account for the normal heterogeneity of the breast and facilitate differentiation between benign and malignant changes in patients undergoing breast conservation therapy for the treatment of breast cancer. Future studies will incorporate these patient demographic variables into the optical assessment to determine which parameters would be best utilized for margin assessment.

## Conclusions

This observational study shows that menopausal status and patient demographics will be a factor in maximizing the diagnostic capabilities of optical spectroscopy for breast cancer margin assessment. The site level analysis demonstrates which variables are best at discriminating cancer against a fatty background and against a fibrous background. The correct variables for margin level assessment can then be chosen after taking patient demographics into account to determine which tissue background will be encountered. We found scattering to be the most effective in post-menopausal women and [β-carotene] or [THb] to be effective in pre-menopausal women.

## Abbreviations

A: adipose; BMI: body mass index; deoxyHb: deoxygenated hemoglobin; DCIS: ductal carcinoma *in situ*; DR: diffuse reflectance; F: fibrous; FA: fibro-adipose; FCC: fibrocystic change; FG: fibro-glandular; FN: fat necrosis; IDC: invasive ductal carcinoma; ILC: invasive lobular carcinoma; LCIS: lobular carcinoma *in situ*; Mx: mixed tissue types; MAD: median absolute deviance; NIR: near infrared spectrum; oxyHb: oxygenated hemoglobin; THb: total hemoglobin; UV: ultra-violet; V: vessels; VIS: visible; λ: optical wavelength; < μ_a _>: mean optical absorption coefficient; < μ_s_' >: mean optical reduced scattering coefficient.

## Competing interests

JQB has received a salary as an employee of Endls Optics, which may gain or lose financially from the publication of this manuscript. MJ has received a salary as an employee of Endls Optics, which may gain or lose financially from the publication of this manuscript. NR is an employee of Endls Optics and holds stock in Endls Optics. She is applying for patents but has not received reimbursements. SK, JG, TB, WB and LW declare that they have no competing interests.

## Authors' contributions

SK helped conceive of the study, participated in the study design and coordination, collected data, performed data analysis and the statistical analysis, and drafted the manuscript. JG participated in the study design, provided histological diagnoses for all data, and contributed to the organization and content of the manuscript. TB participated in the study design, collected intro-operative measurements, performed data analysis, and contributed to the content and organization of the manuscript. JQB participated in the study design, provided feedback on interpreting the data, and contributed to the content of the manuscript. JG acquired patient consent, collected intra-operative measurements, collated patient data, and contributed to revisions of the manuscript. MJ participated in the study design and coordination and helped to organize and revise the manuscript. WB participated in the study design, helped with the statistical and data analysis, and contributed to the organization and content of the manuscript. NR helped conceive of the study, contributed to the study design and coordination, participated in drafting and critically revising the manuscript and figures. LW helped conceive of the study, participated in the study design, provided intro-operative guidance, and contributed to the organization and content of the manuscript. All authors read and approved the final manuscript.

## Authors' information

Stephanie Kennedy and Torre Bydlon are PhD candidates in Biomedical Engineering at Duke University. Research interests include optical spectroscopy, and optical technologies for margin assessment. They work in the Tissue Optical Spectroscopy (TOpS) Lab under the guidance of Dr Nimmi Ramanujam.

Dr Geradts is Professor of Pathology and attending surgical pathologist at Duke University Medical Center. He is an expert in breast pathology.

Dr J Quincy Brown is a Post-Doctoral fellow in Dr. Ramanujam's lab and is interested in the use of optical spectroscopy and imaging tools for the detection and study of cancer.

Jennifer Gallagher works with Dr. Wilke to obtain patient consent and also aids in intra-operative orientation and measurements.

Marlee Junker has experience in biomedical research, technology transfer, submission of regulatory applications to the FDA, and pre-clinical and clinical research. She has also successfully overseen the administration of this project.

WB is Director of the Bioinformatics for the Duke Comprehensive Cancer Institute and an assistant professor in the Department of Biostatistics and Bioinformatics. He provides experience in statistical design and analysis of clinical trials and the incorporation of statistical methods in translational and clinical research.

Dr. Nimmi Ramanujam is an associate professor of Biomedical Engineering at Duke University. Dr. Ramanujam's interests in the field of biophotonics are centered on research and technology development for applications to cancer.

Dr. Wilke was an Associate Professor of Surgery at the Duke University Comprehensive Cancer Center who has recently moved to the University of Wisconsin as the Director of the Breast Center. She is a member of the Executive Committee for ACOSOG and an elected member of the Commission on Cancer. In association with Drs. Ramanujam and Geradts, she has developed a comprehensive translational research program evaluating the use of optical spectroscopy in the diagnosis and management breast cancer.
